# Supplementary material for: Haplotype-Phased Chromosome-Level Genome Assembly of Floccularia luteovirens Provides Insights into Its Taxonomy, Adaptive Evolution, and Biosynthetic Potential
Source: J Fungi (Basel). 2025 Aug 25;11(9):621. doi: 10.3390/jof11090621 (PMC12470553; doi:10.3390/jof11090621)
Supplement: Supplementary file 1 [file jof-11-00621-s001.zip › jof-3762210-supplementary.pdf]

Haplotype-phased Chromosome-level Genome Assembly of *Floccularia luteovirens* Provides Insights into its Taxonomy, Adaptive Evolution, and Biosynthetic Potential

Jianzhao Qi[1, 2, 3, #], Xiu-Zhang Li [2, #], Ming Zhang [1, 3], Yuying Liu [1, 3], Zhen-xin Wang [1, 3], Rui Xing[4], Khassanov Vadim[5], Minglei Li [3*], Yuling Li [2*]

[1] Shaanxi Key Laboratory of Natural Products & Chemical Biology, College of Chemistry & Pharmacy, Northwest A&F University, Yangling 712100, China. qjz@nwafu.edu.cn (J.Q), zhang-ming@nwafu.edu.cn (M. Z.), 3346462730@nwafu.edu.cn (Z. W.)

[2] State Key Laboratory of Plateau Ecology and Agriculture, Qinghai Academy of Animal and Veterinary Sciences, Qinghai University, Xining 810016, China. xiuzhang11@163.com (X. L.), chuyutang0410@163.com (C. T.), yulingli2000@163.com (Y.L.)

[3] Center of Edible Fungi, Northwest A&F University, Yangling 712100, China. mlli@nwafu.edu.cn.com (M.L.)

[4] Northwest institute of plateau biology, the Chinese Academy of Sciences, 23# Xinning Lu, Xining, Qinghai 810008, China. xingrui@nwipb.cas.cn (R. X.)

[5] Department of Plant Protection and Quarantine, Faculty of Agronomy, S. Seifullin Kazakh Agrotechnical University, Zhenis Avenue, Astana 010011, Kazakhstan, vadim_kazgatu@mail.ru (K. V.)

[#] These authors contributed equally to this work.

* Correspondence: yulingli2000@163.com (Y.L.), mlli@nwafu.edu.cn.com (M.L.);

Content

Table S1. Statistics of PacBio Sequel II sequencing data mapping of *F. luteovirens* QHU-1 genome. 1
Table S2. Statistics of de novo sequencing data mapping of *F. luteovirens* QHU-1 genome. 2
Table S3. Statistics of Hi-C sequencing data mapping of *F. luteovirens* QHU-1 genome. 3
Table S4. Estimation of genome size of *F. luteovirens* QHU-1. 4
Table S5. Statistical table of *F. luteovirens* QHU-1 assembly results. 5
Table S6. Statistical table of the length of the assembly sequence of *F. luteovirens* QHU-1. 6
Table S7. Statistics of BUSCO evaluation of *F. luteovirens* QHU-1 genome. 7
Table S8. Genetic Information the Statistical Table of protein-coding genes. 8
Table S9. Statistics of non-coding RNA annotation results in *F. luteovirens* QHU-1 genome. 9
Table S10. Statistics of *F. luteovirens* QHU-1 protein-coding gene annotation. 11
Table S11. Statistics of *F. luteovirens* QHU-1 repetitive sequence annotation results. 12
Table S12. Statistics for SNP of *F. luteovirens* QHU-1. 13
Table S13. Tandem repeat results statistics of *F. luteovirens* QHU-1. 14
Table S14. The results of the alignment of *F. luteovirens* QHU-1 terpenoid synthase. 15
Table S15. Protein sequence information used to construct the evolutionary tree of *F. luteovirens* QHU-1 sesquiterpene synthase. 16
Table S16. Protein sequence information used to construct the evolutionary tree of *F. luteovirens* QHU-1 PKS. 18
Figure S1. Kmer-Depth and Kmer Species-Frequency Distribution Plot. 19
Figure S2. The GC depth of haplotype A of the genome of *F. luteovirens* QHU-1. 20
Figure S3. The GC depth of haplotype B of the genome of *F. luteovirens* QHU-1. 20
Figure S4. Interaction matrices constructed at the genome-wide level of haplotype A of *F. luteovirens* QHU-1. 21
Figure S5. Interaction matrices constructed at the genome-wide level of haplotype B of *F. luteovirens* QHU-1. 21
Figure S6. Statistical Chart of KOG Functional Annotated Classification of haplotype A of *F. luteovirens* QHU-1 genome. 22
Figure S7. Statistical Chart of KOG Functional Annotated Classification of haplotype B of *F. luteovirens* QHU-1 genome. 22
Figure S8. KEGG Pathway Functional Classification Chart of haplotype A of *F. luteovirens* QHU-1 genome. 23
Figure S9. KEGG Pathway Functional Classification Chart of haplotype B of *F. luteovirens* QHU-1 genome. 23
Figure S10. Statistical map of functional annotation classification based on GO database of haplotype A of *F. luteovirens* QHU-1 genome. 24
Figure S11. Statistical map of functional annotation classification based on GO database of haplotype B of *F. luteovirens* QHU-1 genome. 24
Figure S12. GO, NR, SWISS, KEGG, COG Venn diagram of haplotype A of *F. luteovirens* QHU-1 genome. 25
Figure S13. GO, NR, SWISS, KEGG, COG Venn diagram of haplotype B of *F. luteovirens* QHU-1 genome. 25
Figure S14. Ka curves for *F. luteovirens* QHU-1 and two related fungi. 26
Figure S15. Ks curves for *F. luteovirens* QHU-1 and two related fungi. 26

Table S1. Statistics of PacBio Sequel II sequencing data mapping of *F. luteovirens* QHU-1 genome.

Item	Value
Reads Number	10,192,574
Reads Bases (bp)	59,372,423,888
Largest Length (bp)	303,062
N50 Length (bp)	6,569
N90 Length (bp)	4,225
Average Length (bp)	5,825

Table S2. Statistics of de novo sequencing data mapping of *F. luteovirens* QHU-1 genome.

Item	Value
Insert size	450 bp
Raw data	5,644.4Mb
Clean data	5,570.3 Mb
Clean data Q20	97.70%
Clean data Q30	94.55%
Clean data GC	42.73%

Table S3. Statistics of Hi-C sequencing data mapping of *F. luteovirens* QHU-1 genome.

Item	Value
Insert size	450 bp
Raw data	2,725.5 Mb
Clean data	2,684.4 Mb
Clean data Q20	98.10%
Clean data Q30	94.87%
Clean data GC	42.89%

Table S4. Estimation of genome size of *F. luteovirens* QHU-1.

Item	Number
kmer	21
Genome size	27.7M
Heterozygosity	1.36%
Repeat	1.34%

Genome size was estimated using genomescope v2.0 (http://genomescope.org/genomescope2.0/) software.

Table S5. Statistical table of *F. luteovirens* QHU-1 assembly results.

Item	Value	
	Haplotype A	Haplotype B
Total Length	26,770,180	27,037,179
N50 length	2,344,500	2,323,414
N90 length	1,381,188	1,411,032
GC Content (%)	43.54%	43.52%
N rate (%)	0	0

Table S6. Statistical table of the length of the assembly sequence of *F. luteovirens* QHU-1.

Chr	Length	Nrate	GC
Chr1	3271583	0%	44.02%
Chr2	3181987	0%	43.58%
Chr3	2803076	0%	43.91%
Chr4	2767490	0%	43.76%
Chr5	2323414	0%	43.50%
Chr6	2009781	0%	43.72%
Chr7	1983262	0%	43.73%
Chr8	1718499	0%	43.15%
Chr9	1479638	0%	42.88%
Chr10	1448046	0%	43.00%
Chr11	1411032	0%	42.94%
Chr12	1321638	0%	43.12%
Chr13	1279218	0%	43.6%
Chr14	38515	0%	22.45%

Table S7. Statistics of BUSCO evaluation of *F. luteovirens* QHU-1 genome.

Item	Number	Percent (%)
Complete BUSCOs (C)	740	97.6
Complete and single-copy BUSCOs (S)	739	97.5
Complete and duplicated BUSCOs (D)	1	0.1
Fragmented BUSCOs (F)	5	0.7
Missing BUSCOs (M)	13	1.7
Total BUSCO groups searched (n)	758	100.0

Single-copy for single-copy BUSCOs; duplicated for multicopy BUSCOs; Fragmented for fragmented BUSCOs; Missing for missing BUSCOs. The predicted genes were assessed for completeness using the BUSCO software (version: 5.3.2) based on the fungi database (fungi_odb10).

Table S8. Genetic Information the Statistical Table of protein-coding genes.

Item	Number	
	Haplotype A	Haplotype B
Genome size(bp)	26,770,180	27,037,179
Gene number	4,552	4,595
Gene total length(bp)	7,321,347	7,352,842
Gene average length(bp)	1,608	1,600
Gene length/Genome(%)	27.35	27.20

Table S9. Statistics of non-coding RNA annotation results in *F. luteovirens* QHU-1 genome.

Sample	Class		number	totalLen(bp)	meanLen(bp)	% in Genome
Haplotype A	tRNA		101	84	22,899	0.0320
	sRNA		0	0	0	0
	snRNA		7	138	966	0.0036
	miRNA		0	0	0	0
	rRNA_de	5S	4	114	456	0.0616
		5.8S	0	0	0	
		18S	3	1,808	5,424	
		28S	3	3,540	10,620	
	rRNA_ho	5S	-	-	-	-
		5.8S	-	-	-	
		18S	-	-	-	
		28S	-	-	-	
Haplotype B	tRNA		98	84	8,311	0.0307
	sRNA		0	0	0	0
	snRNA		7	138	966	0.0036
	miRNA		0	0	0	0
	rRNA_de	5S	5	114	570	0.1119
		5.8S	0	0	0	
		18S	5	1,808	9,040	

	28S	5	4,127	20,636	
rRNA_ho	5S	-	-	-	-
	5.8S	-	-	-	
	18S	-	-	-	
	28S	-	-	-	

rRNA is ribosomal RNA; tRNA is transport RNA; sRNA is small regulatory RNA; snRNA is nucleolar small RNA. **totalLen** and **meanLen** are the total length and mean length.

Table S10. Statistics of *F. luteovirens* QHU-1 protein-coding gene annotation.

Sample	Item	Count	Percentage
Haplotype A	All	4,545	100%
	Annotation	4,325	95.16%
	Swiss	2,705	59.52%
	Nr	4,325	95.16%
	GO	1,934	42.55%
	KEGG	1,726	37.98%
	COG	2,951	64.93%
Haplotype B	All	4,591	100%
	Annotation	4,364	95.06%
	Swiss	2,747	59.83%
	Nr	4,364	95.06%
	GO	1,951	42.50%
	KEGG	1,729	37.66%
	COG	2,966	64.60%

Annotation is the gene with at least one annotation; Uniprot is the gene annotated to the Uniprot database; Nr is the gene that is annotated to the Nr database; Interproscan is the gene that is annotated to the Interproscan GO is the gene annotated to the GO database; KEGG is the gene that is annotated to the KEGG database; Pathway is the gene that is annotated to the KEGG Pathway database; COG is the gene that is annotated to the COG database.

Table S11. Statistics of *F. luteovirens* QHU-1 repetitive sequence annotation results.

Type	number	totalLen(bp)	meanLen(bp)	In Genome (%)
LTR	2,565	1,104,556	439	4.0853
DNA	682	108,690	162	0.4020
LINE	556	56,154	103	0.2077
SINE	5	324	65	0.0012
RC	83	16,086	194	0.0595
scRNA	0	0	0	0
Unknown	3,572	1,493,142	436	5.5226
Total	7,463	2,746,507	384	10.1583

Table S12. Statistics for SNP of *F. luteovirens* QHU-1.

No.	Number	
	Haplotype A	Haplotype B
Chr1	32995	32346
Chr2	34953	36774
Chr3	29100	29762
Chr4	28386	28293
Chr5	29694	28266
Chr6	24322	24851
Chr7	24185	24706
Chr8	26734	24837
Chr9	21770	22173
Chr10	24746	24644
Chr11	23555	22928
Chr12	22572	21565
Chr13	21834	21175
Chr14	9	10
Total	344,855	342,330

Table S13. Tandem repeat results statistics of *F. luteovirens* QHU-1.

Type	number	Repeat Size(bp)	Total Length(bp)	In Genome (%)
TRF	2,324	1~802	121,447	0.4492
Minisatellite DNA	1,997	10~60	92,464	0.3420
Microsatellite DNA	133	2~6	8,576	0.0317

Table S14. The results of the alignment of *F. luteovirens* QHU-1 terpenoid synthase.

Gene ID	Anotation	Identities	Source	Accession number
1001698.1	terpenoid synthase	82.66	*Neolentinus lepideus* HHB14362 ss-1	KZT23360.1
1002574.1	isoprenoid synthase	44.95%	*Crucibulum laeve*	TFK39619.1
1002765.1	isoprenoid synthase	60.43%	*Infundibulicybe gibba*	KAF8889626.1
1002832.1	squalene synthase	83.29%	*Pholiota molesta*	KAF8194667.1
1003064.1	isoprenoid synthase	70.57%	*Cyathus striatus*	KAF9000618.1
1003593.1	isoprenoid synthase	38.71%	*Infundibulicybe gibba*	KAF8883523.1
1004092.1	isoprenoid synthase	86.50%	*Crassisporium funariophilum*	KAF8155792.1

Table S15. Protein sequence information used to construct the evolutionary tree of *F. luteovirens* QHU-1 sesquiterpene synthase.

NO.	Gene Name	Accession number
1	STC4	KAH0582448
2	STC9	KAH0583476
3	STC15	KAG5341349
4	Cop1	XP_001832573
5	Cop2	XP_001836556
6	Cop3	XP_001832925
7	Cop4	XP_001836356
8	Cop5	XP_001834007
9	Cop6	XP_001832549
10	AcTPS4	M_Fcontig40411
11	AcTPS5	M_Fcontig40579
12	AcTPS9	M_Fcontig47706
13	CpSTS1	LC436345.1
14	CpSTS2	LC436346.1
15	CpSTS3	LC436347.1
16	CpSTS4	LC436348.1
17	CpSTS5	LC436349.1
18	CpSTS6	LC436350.1
19	CpSTS7	LC436351.1
20	CpSTS8	LC436352.1
21	CpSTS9	LC436353.1
22	CpSTS10	LC436354.1
23	CpSTS11	LC436355.1
24	CpSTS12	LC436356.1
25	CpSTS13	LC436357.1
26	CpSTS14	LC436358.1
27	CpSTS15	LC436359.1
28	CpSTS16	LC436360.1
29	CpSTS17	LC436361.1
30	CpSTS18	LC436362.1
31	Hfas94a	MK287936.1
32	Hfas94b	MK287937.1
33	Hfas344	MK287938.1
34	Hypsu1_138665	Hypsu1_138665
35	Agr1	MN146024
36	Agr2	MN146025
37	Agr3	MN146026
38	Agr4	MN146027
39	Agr5	MN146028
40	Agr6	MN146029

41	Agr7	MN146030
42	Agr8	MN146031
43	Agr9	MN146032
44	Agr10	MN146033
45	Agr11	MN146034
46	Pro1	AGR34199
47	Galma_104215	Galma_104215
48	Omp1	1311
49	Omp3	4636
50	Omp4	1447
51	Omp5a	2392
52	Omp5b	2393
53	Omp6	4774
54	Omp7	2271
55	Omp8	
56	Omp9	3258
57	Omp10	3981
58	1001698.1	
59	1002574.1	
60	1002765.1	
61	1003064.1	
62	1003593.1	
63	1004092.1	

Table S16. Protein sequence information used to construct the evolutionary tree of *F. luteovirens* QHU-1 PKS.

No.	Name	Accession number
1	HerA	
2	ArmB	I3ZNU9
3	CrPKS1	OQ863313
4	CrPKS2	OQ863314
5	CrPKS3	OQ863315
6	CoPKS1	OL512945
7	CoPKS4	OL512946
8	PKS2	APH07628
9	PKS15	ESK96613
10	PKS1	APH07629
11	PKS63787	KX683290
12	CC1G_05377	XP_001835415

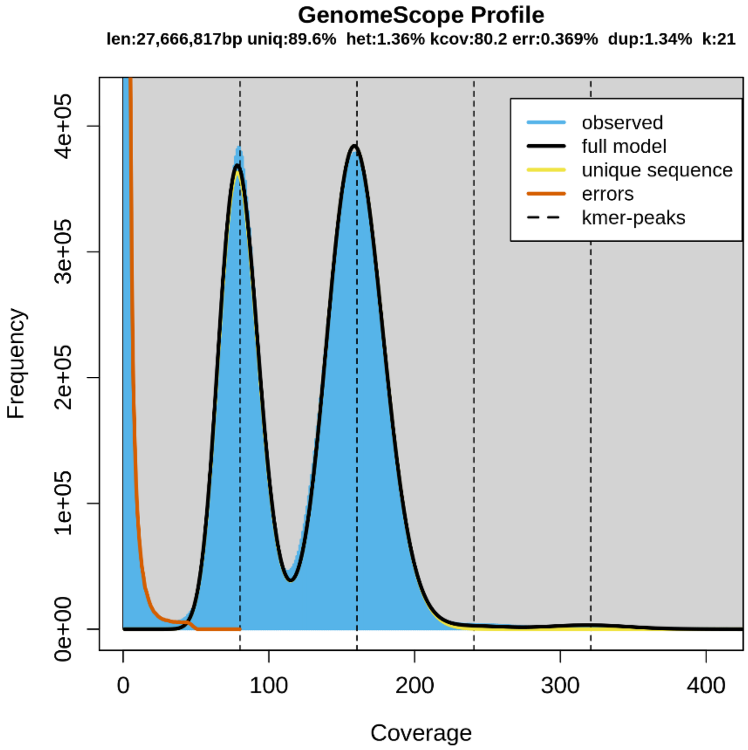

Figure S1. Kmer-Depth and Kmer Species-Frequency Distribution Plot.

The blue line represents the actual K-mer curve, the black line is the k-mer curve estimated by the model, the yellow line is the K-mer curve corresponding to the unique data, the red line represents the error curve due to sequencing errors, and the dashed line represents speculation K-mer peak.

Using the reads obtained by sequencing, K-mer-based analysis was used to estimate the genome size and heterozygosity. A K-mer refers to a sequence of K bp in length. Iteratively select a sequence of length K bases from a continuous sequence. If the length of the sequence is L and the length of the K-mer is K, then L-K+1 K-mers can be obtained. We take K-mers for the reads obtained by sequencing, and then count the frequency of each K-mer. According to the Lander waterman algorithm, the genome size (G) satisfies the following formula:

$$C_{base} = C_{k-mer} \times \frac{L}{L-K+1}$$

$$G = \frac{n_{k-mer}}{C_{k-mer}} = \frac{n_{base}}{C_{base}}$$

C_{base} and $C_{k\text{-mer}}$ are the expected depth of coverage and K-mer, and n base and n k-mer are the total number of bases and the total number of K-mers in the sequence. In the case of a certain amount of data, the depth frequency of K-mer is subject to Poisson distribution, so the peak of the K-mer depth frequency distribution is the corresponding depth, which is used as an estimate of the expected depth of K-mer.

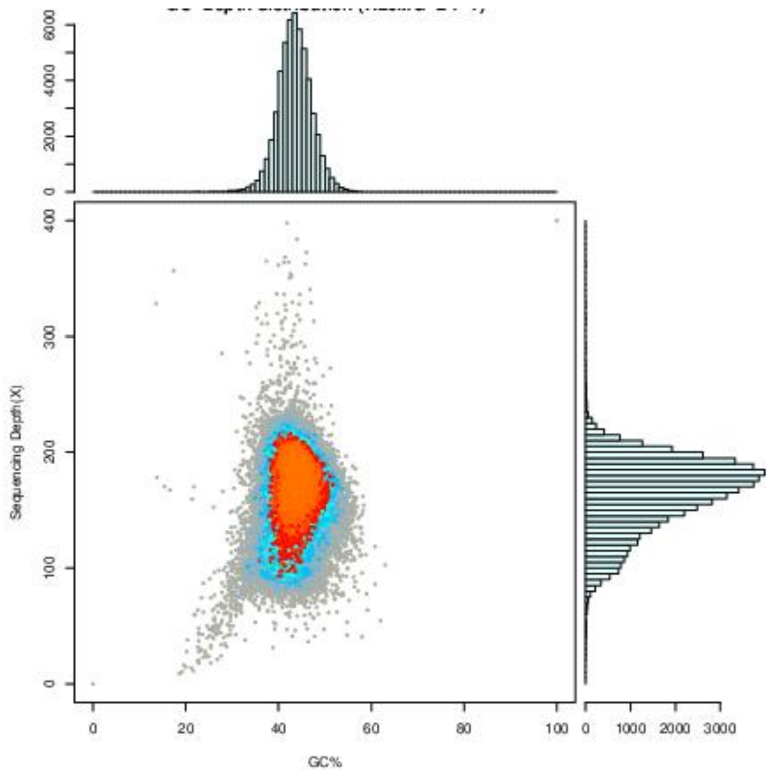

Figure S2. The GC depth of haplotype A of the genome of *F. luteovirens* QHU-1.

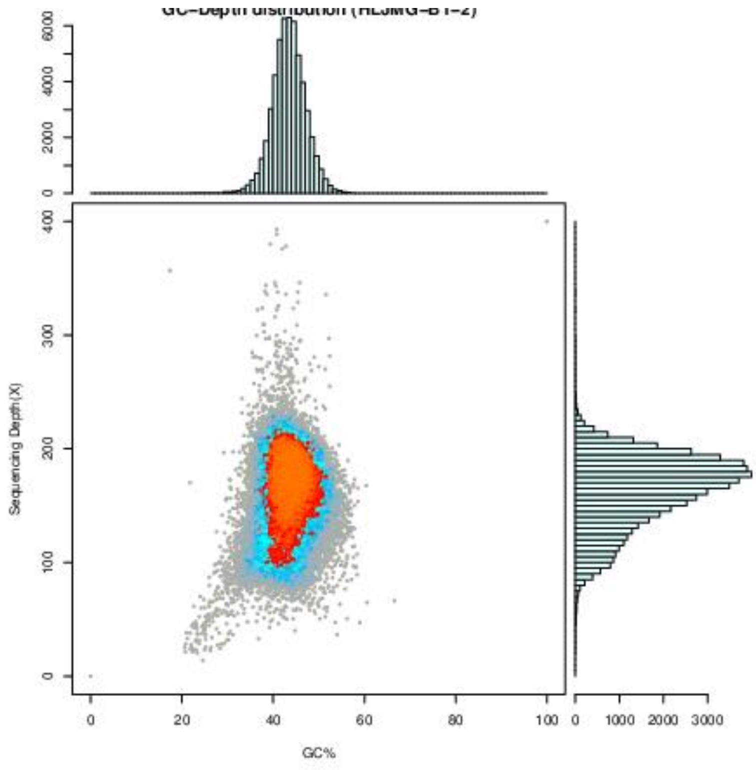

Figure S3. The GC depth of haplotype B of the genome of *F. luteovirens* QHU-1.

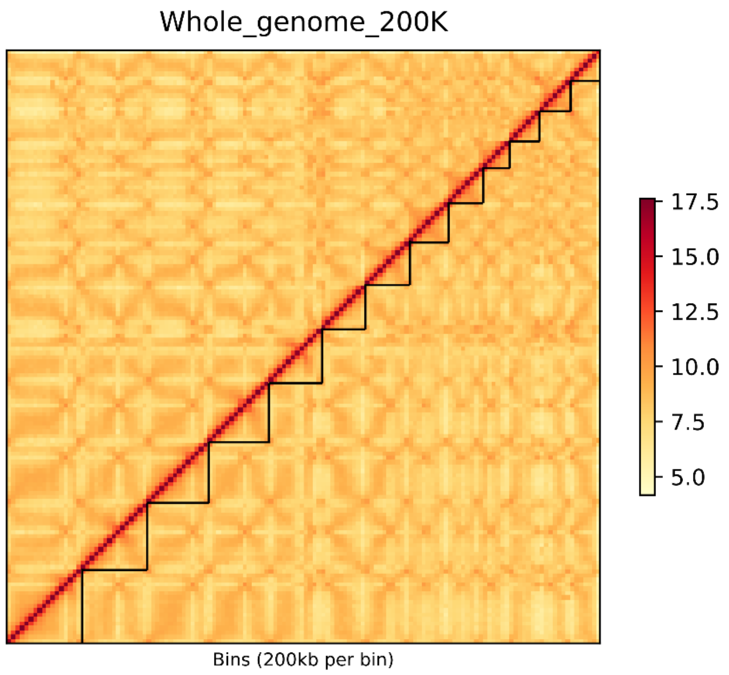

Figure S4. Interaction matrices constructed at the genome-wide level of haplotype A of *F. luteovirens* QHU-1.

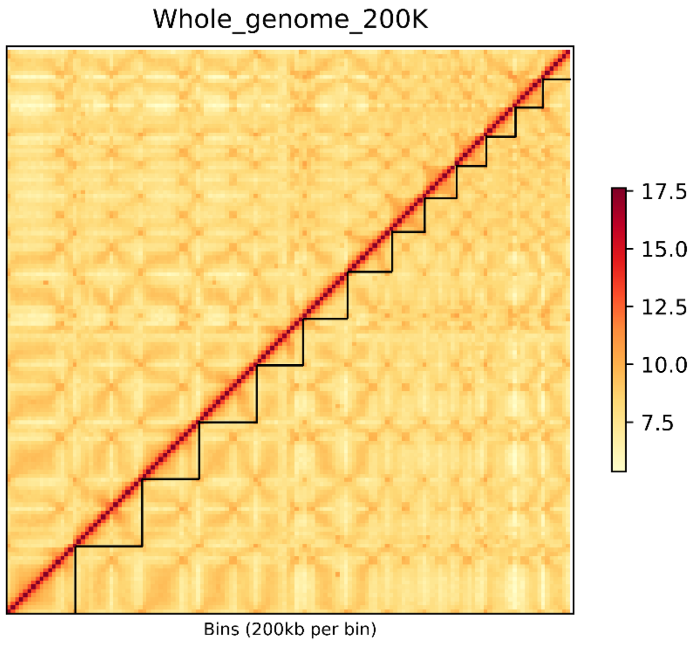

Figure S5. Interaction matrices constructed at the genome-wide level of haplotype B of *F. luteovirens* QHU-1.

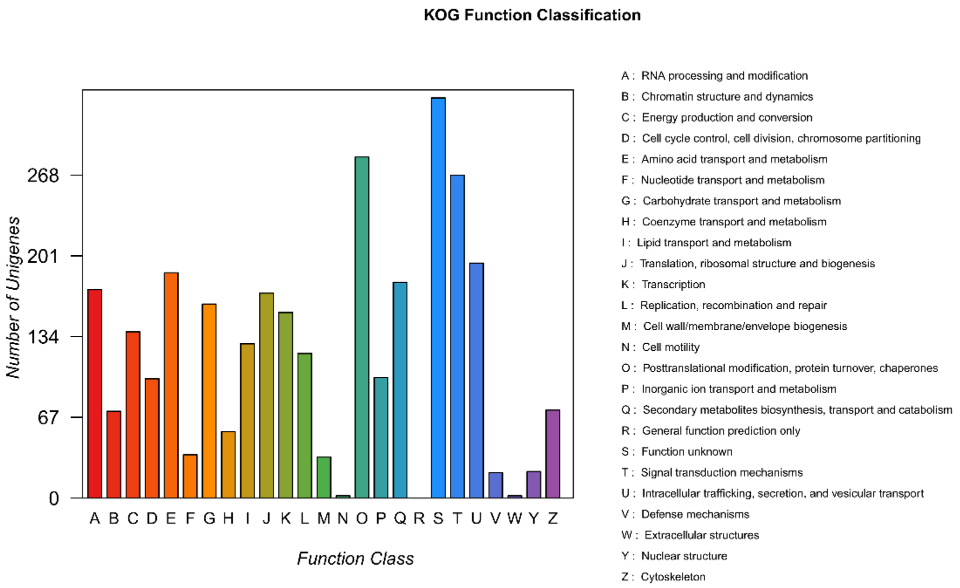

Figure S6. Statistical Chart of KOG Functional Annotated Classification of haplotype A of *F. luteovirens* QHU-1 genome.

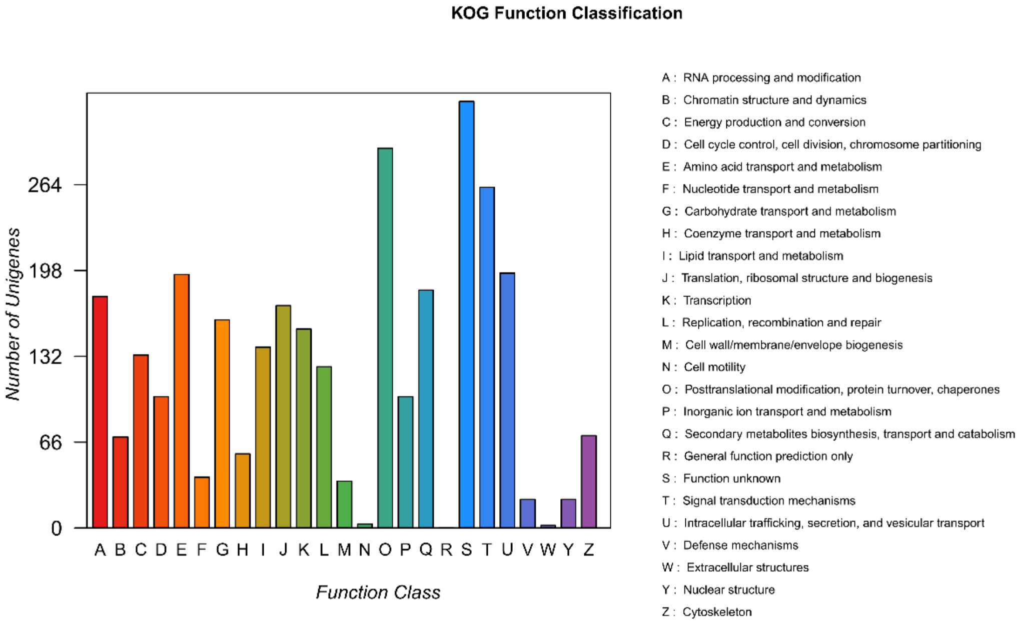

Figure S7. Statistical Chart of KOG Functional Annotated Classification of haplotype B of *F. luteovirens* QHU-1 genome.

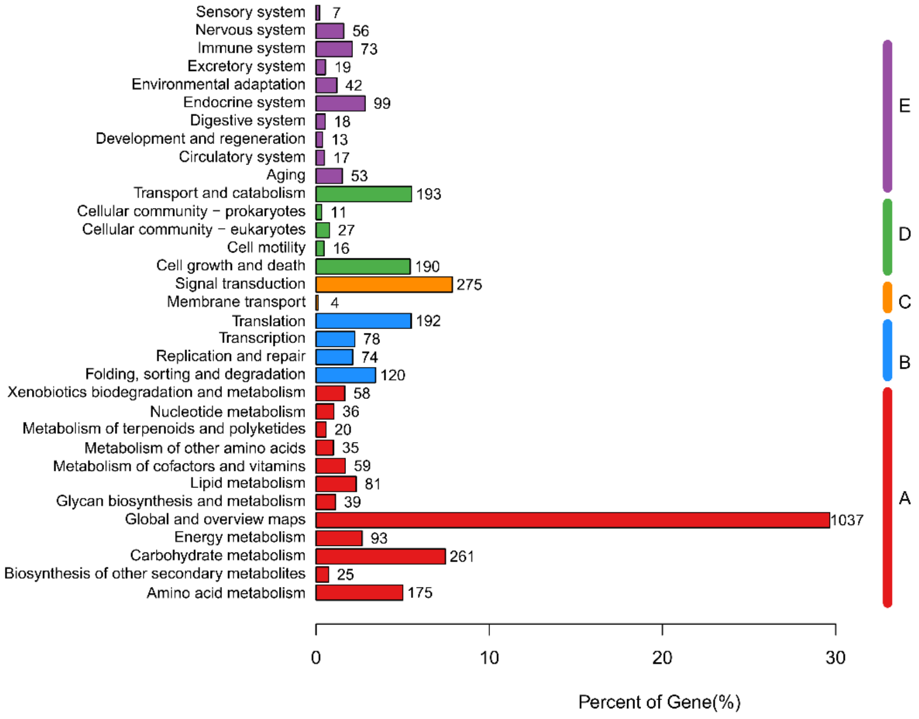

Figure S8. KEGG Pathway Functional Classification Chart of haplotype A of *F. luteovirens* QHU-1 genome.

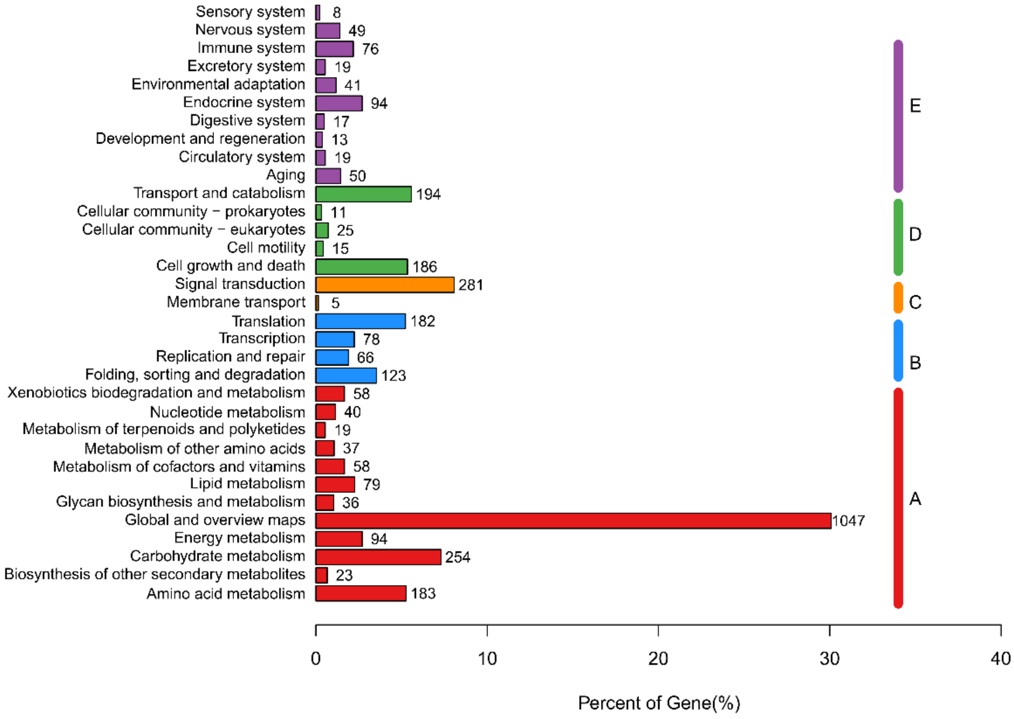

Figure S9. KEGG Pathway Functional Classification Chart of haplotype B of *F. luteovirens* QHU-1 genome.

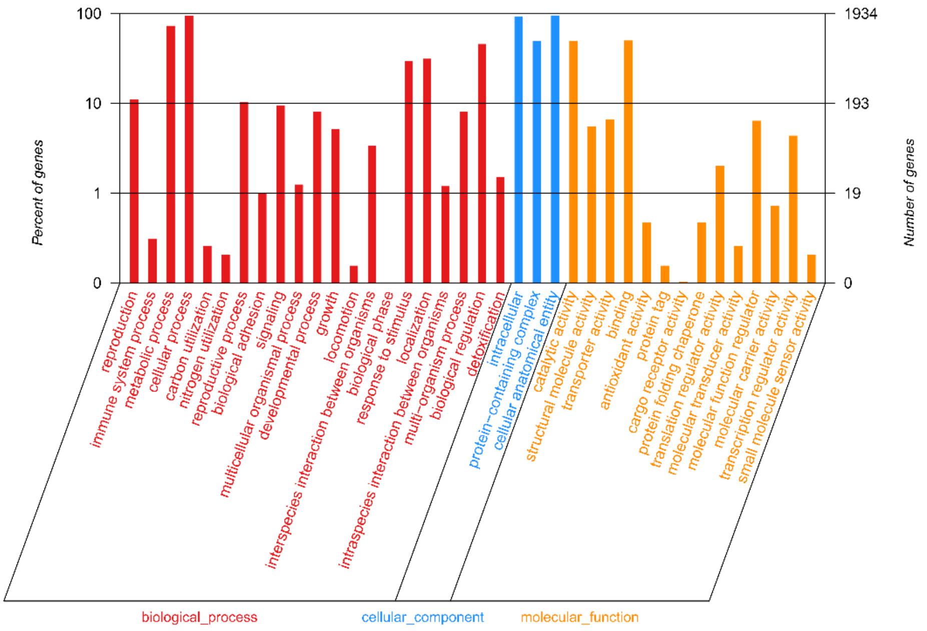

Figure S10. Statistical map of functional annotation classification based on GO database of haplotype A of *F. luteovirens* QHU-1 genome.

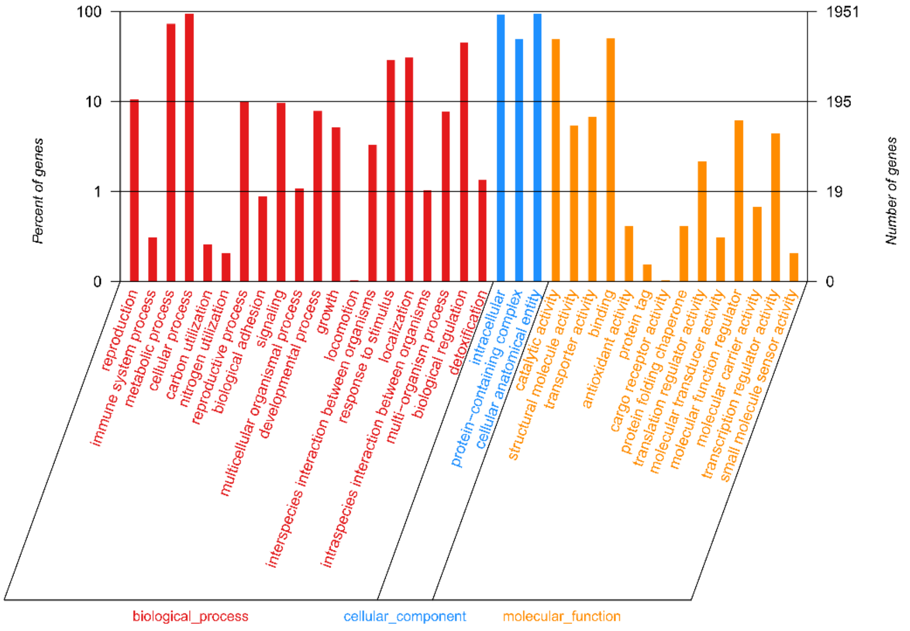

Figure S11. Statistical map of functional annotation classification based on GO database of haplotype B of *F. luteovirens* QHU-1 genome.

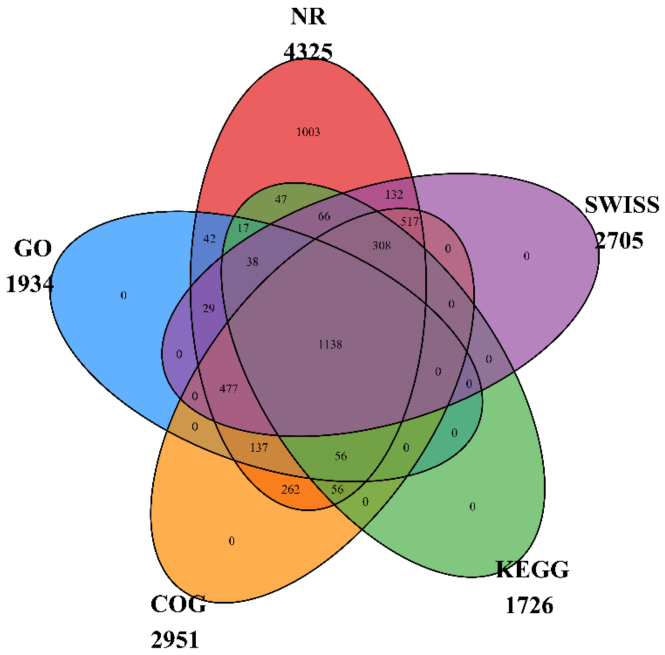

Figure S12. GO, NR, SWISS, KEGG, COG Venn diagram of haplotype A of *F. luteovirens* QHU-1 genome.

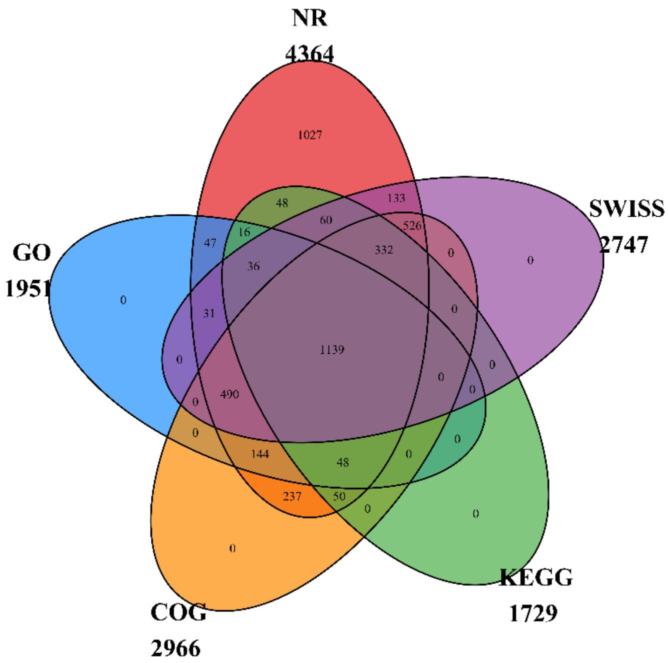

Figure S13. GO, NR, SWISS, KEGG, COG Venn diagram of haplotype B of *F. luteovirens* QHU-1 genome.

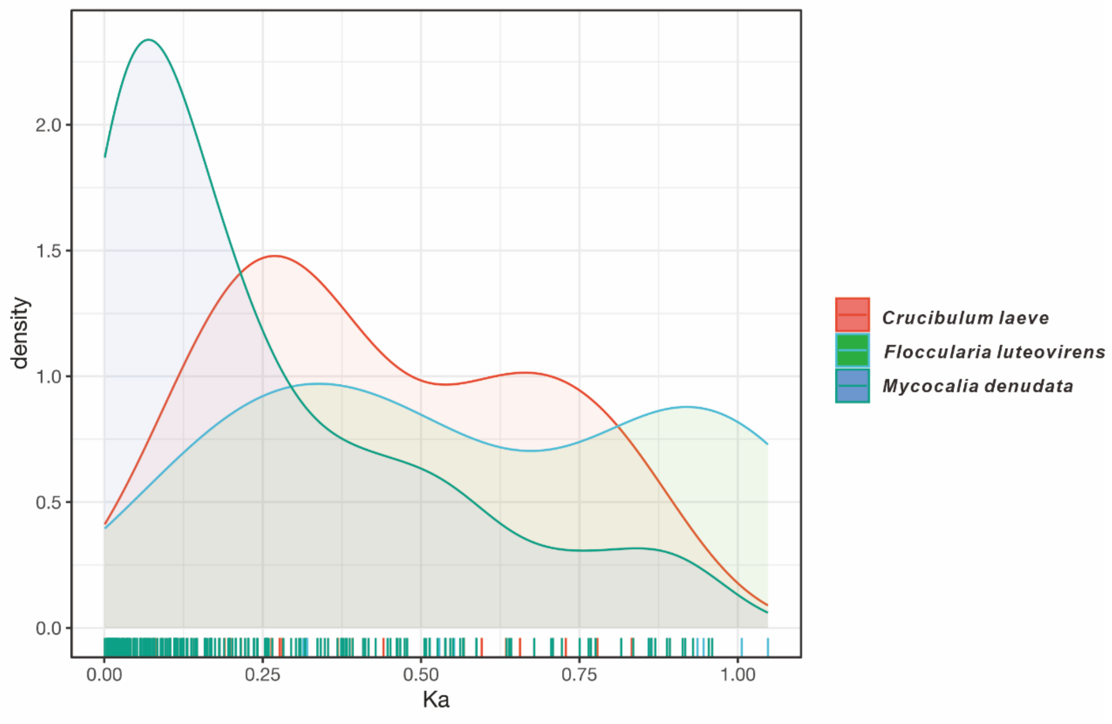

Figure S14. Ka curves for *F. luteovirens* QHU-1 and two related fungi.

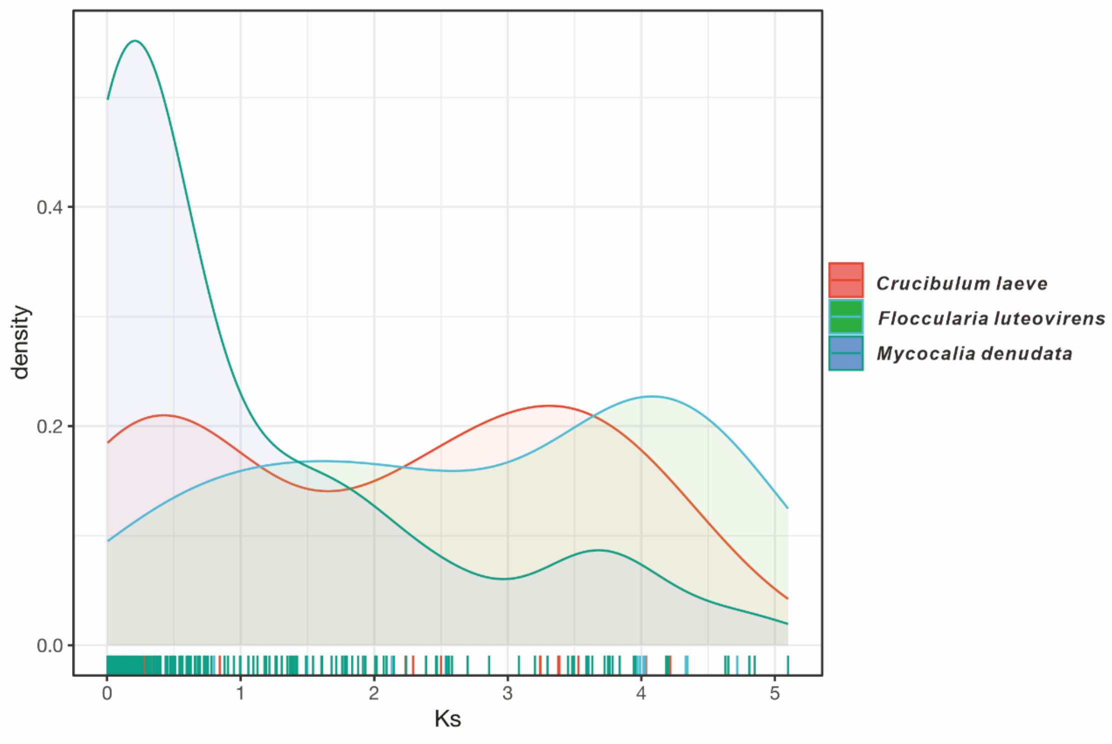

Figure S15. Ks curves for *F. luteovirens* QHU-1 and two related fungi.
